# Existential Protest and Behavioural Resistance During Pandemics: Ontological Reckoning, Psychodynamic Conflict, and the Samos Syndrome

**DOI:** 10.1192/j.eurpsy.2026.11887

**Published:** 2026-07-31

**Authors:** C. Lazzari

**Affiliations:** Psychiatry, International Centre for Healthcare and Medical Education (ICHME), London, United Kingdom

## Abstract

**Introduction:**

Pandemics fracture not only biological and institutional systems but also the ontological integrity of human experience. Resistance to preventive behaviours often reflects an existential protest—a refusal to accept vulnerability, impermanence, and proximity to death. Crisis thus operates as a site of ontological reckoning, revealing tensions between Eros and Thanatos, intimacy and withdrawal, control and surrender.

**Objectives:**

To investigate behavioural resistance in pandemic contexts as a psychodynamic and existential phenomenon. The study examines unconscious motives, symbolic enactments, and relational vulnerability, exploring how actions during crises reflect complex ontological and emotional tensions rather than simple irrationality or neglect.

**Methods:**

A philosophical–psychoanalytic approach was adopted, drawing on clinical literature, phenomenology, and psychoanalytic theory. Textual sources include accounts from HIV/AIDS and COVID-19 crises, exploring constructs such as irrationalism, solipsism, altruistic surrender, and the Samos Syndrome. The interpretive focus was placed on affective ambivalence, unconscious desire, and symbolic meaning in health-risking behaviors.

**Results:**

Critical incidents revealed intricate psychodynamic narratives underpinning pandemic-era decisions. Examples include individuals who engaged knowingly in unprotected sex with high-risk partners, framing such acts as relational commitment or sacrificial love. Resistance to protective measures often emerged through unconscious conflict, preconscious impulses, or existential disavowal. Behaviour patterns reflected disruptions in self–other boundaries and ontological coherence, challenging conventional prevention frameworks.

**Table 1.** Table 1. long description.

Section	Key Concepts	Analytical Insights	Implications
**Results**	Psychodynamic conflict; sacrificial intimacy; existential disavowal; Samos Syndrome	Behavioural resistance stems from unconscious motives and symbolic enactments that defy epidemiological logic	Highlights instability in self–other boundaries and ontological incoherence within health-risk behaviours
**Conclusions**	Ontological distress; symbolic reasoning; affective vulnerability	Pandemic actions function as expressions of existential reckoning and emotional protest	Calls for integrative public health models grounded in narrative, phenomenology, and psychodynamic insight

**Conclusions:**

Pandemic resistance behaviours are shaped by symbolic reasoning, psychodynamic vulnerability, and existential distress. These enactments defy purely rational intervention and call for psychiatric models that integrate phenomenological depth, narrative understanding, and ontological sensitivity. Prevention strategies must engage with the emotional architecture of crisis, recognising the lived complexity of human behaviour under threat.

**Disclosure of Interest:**

None Declared

## Long descriptions

### Table 1. Long description

The table consists of four columns: Section, Key Concepts, Analytical Insights, and Implications.

Row 1 (Results):

- Key Concepts: Psychodynamic conflict, sacrificial intimacy, existential disavowal, and Samos Syndrome.

- Analytical Insights: Behavioural resistance stems from unconscious motives and symbolic enactments that defy epidemiological logic.

- Implications: Highlights instability in self-other boundaries and ontological incoherence within health-risk behaviours.

Row 2 (Conclusions):

- Key Concepts: Ontological distress, symbolic reasoning, and affective vulnerability.

- Analytical Insights: Pandemic actions function as expressions of existential reckoning and emotional protest.

- Implications: Calls for integrative public health models grounded in narrative, phenomenology, and psychodynamic insight.

Navigate back to Table 1..

